# Review on Multicomponent Hydrogel Bioinks Based on Natural Biomaterials for Bioprinting 3D Liver Tissues

**DOI:** 10.3389/fbioe.2022.764682

**Published:** 2022-02-14

**Authors:** Daekeun Kim, Minseok Kim, Jongwan Lee, Jinah Jang

**Affiliations:** ^1^ Department of Convergence IT Engineering, Pohang University of Science and Technology (POSTECH), Pohang, South Korea; ^2^ Department of Mechanical System Engineering, Kumoh National Institute of Technology, Gumi, South Korea; ^3^ Department of Aeronautics, Mechanical and Electronic Convergence Engineering, Kumoh National Institute of Technology, Gumi, South Korea; ^4^ School of Interdisciplinary Bioscience and Bioengineering, Pohang University of Science and Technology (POSTECH), Pohang, South Korea; ^5^ Department of Mechanical Engineering, Pohang University of Science and Technology (POSTECH), Pohang, South Korea; ^6^ Institute of Convergence Science, Yonsei University, Seoul, South Korea

**Keywords:** tissue engineering, 3D bioprinting, bioink, biomaterial, hydrogel, 3D-bioprinted liver, hepatic regeneration

## Abstract

Three-dimensional (3D)-printed *in vitro* tissue models have been used in various biomedical fields owing to numerous advantages such as enhancements in cell response and functionality. In liver tissue engineering, several studies have been reported using 3D-printed liver tissue models with improved cellular responses and functions in drug screening, liver disease, and liver regenerative medicine. However, the application of conventional single-component bioinks for the printing of 3D *in vitro* liver constructs remains problematic because of the complex structural and physiological characteristics of the liver. The use of multicomponent bioinks has become an attractive strategy for bioprinting 3D functional *in vitro* liver tissue models because of the various advantages of multicomponent bioinks, such as improved mechanical properties of the printed tissue construct and cell functionality. Therefore, it is essential to review various 3D bioprinting techniques and multicomponent hydrogel bioinks proposed for liver tissue engineering to suggest future directions for liver tissue engineering. Accordingly, we herein review multicomponent bioinks for 3D-bioprinted liver tissues. We first describe the fabrication methods capable of printing multicomponent bioinks and introduce considerations for bioprinting. We subsequently categorize and evaluate the materials typically utilized for multicomponent bioinks based on their characteristics. In addition, we also review recent studies for the application of multicomponent bioinks to fabricate *in vitro* liver tissue models. Finally, we discuss the limitations of current studies and emphasize aspects that must be resolved to enhance the future applicability of such bioinks.

## 1 Introduction

Recently, many scholars have attempted to utilize three-dimensional (3D) printing technology to reconstruct *in vitro* tissue models with living organisms (i.e., cells) and have eventually fabricated transplantable substrates for the various organs in the human body for regeneration. During the early stages of applying 3D printing to bio- and tissue engineering, 3D-printed structures were fabricated for the cultivation of cells outside the body (i.e., *in vitro*), with stable environmental conditions for the cells (i.e., 3D scaffolds) (Seol et al., 2014; Zhu et al., 2016; Jang et al., 2018). However, the necessity of reconstructing *in vitro* models, which emulate *in vivo* structures, has been emphasized because of the significantly different mismatches in cell behaviors between *in vivo* and *in vitro* environments (Agarwal et al., 2019). For instance, the morphologies of cells grown in a two-dimensional (2D) environment are different from those of cells grown *in vivo*, thus affecting the cellular processes, including proliferation, differentiation, and protein and gene expressions; this, in turn, results in a different reactivity than that *in vivo* (Edmondson et al., 2014). More recently, as an effective solution, various 3D-printed structures have been introduced for the *in vitro* study of cells. The development of 3D-printed structures is subject to the provision of natural tissue-like environments to the cells (Pati et al., 2014). Therefore, the various factors that affect cells should be considered carefully (Jang et al., 2018). For instance, the fabrication method employed in 3D printing technology needs to be selected carefully to fabricate microstructures in a convenient and effective manner. In addition, the properties and composition of the printed materials play crucial roles in their application, and the mechanical properties and compositions of chemicals significantly affect the functionality and viability of cells (Kyle et al., 2017; Ma et al., 2018). To conveniently address these considerations, cell-laden printable materials were employed for 3D printing bio-applications. Various materials that encapsulate cells were initially handled in the liquid phase (i.e., bioinks) but were solidified subsequently (or during printing) by various stimuli, such as heat, light, pH, and ionic species (Cui et al., 2020). After printing, bioinks should provide a stable, cytocompatible environment to cells until the development of tissues. In this manner, it is important to develop bioinks that can achieve both targeted tissue-specific microenvironments and high printability (Jang et al., 2018; Cui et al., 2020). However, single-component bioinks suffer from limitations in terms of satisfying both the printability and biocompatibility requirements. Therefore, various types of multicomponent bioinks have been developed to replicate the functions of the tissue-specific extracellular matrix (ECM); these have also been applied in the fabrication of 3D functional tissue models for targeted tissue regeneration, drug screening platforms, and disease models, such as the brain, liver, and pancreas. In particular, the liver is known to be one of the most challenging organs to be replicated by 3D-printed bioinks because it is a structurally and physiologically complex organ that consists of various types of cells (Bhatia, 2016; Ma et al., 2020). Notably, hepatocytes cultured in 2D monolayers exhibit different phenotypes than those *in vivo* and have limitations in maintaining long-term functionality and viability. The advent of 3D cell culture technology has overcome these limitations. 3D cell culture systems can recapitulate the *in vivo* microenvironment, including the cell–cell and cell–ECM interactions. In this context, primary human hepatocytes cultured in a 3D environment showed *in vivo-like* morphology and maintained functionality; they also survived for longer periods, as compared to those cultured in a 2D environment (Lauschke et al., 2019). Therefore, 3D *in vitro* liver models have been adopted for engineering functional tissues for implantation, disease pathogenesis studies, and drug screening; these efforts have resulted in significant developments in liver tissue engineering. Various 3D *in vitro* liver tissue models exist, such as pre-fabricated scaffold-based (Jiankang et al., 2009; Lee et al., 2017a; Wu et al., 2019), 3D-printed (Ma et al., 2016; Kang et al., 2018; Kang et al., 2020; Yang et al., 2021), scaffold-free (Kizawa et al., 2017), and livers on-chip (Bhise et al., 2016; Lee and Cho, 2016). Pre-fabricated scaffold-based tissue models could provide cells with a mechanically stable environment (Jiankang et al., 2009; Lee et al., 2017a; Rajendran et al., 2017; Wu et al., 2019). In particular, the scaffold fabricated from the inverted colloidal system (ICC) has homogeneous pores all over the scaffold, which enhances the interaction between cells and the diffusion of oxygen and nutrients to cells (Lee et al., 2017a; Shao et al., 2019; Wu et al., 2019). However, some disadvantages exist in requiring an additional cell seeding process during the manufacturing process and the random distribution of cells over the pre-fabricated scaffold. Also, the production of heterogeneous tissue constructs using pre-fabricated scaffold-based tissue models is challenging. As scaffold-free printing does not require bioinks, the manufacturing process is straightforward and almost completely avoids inducing cell damage. Also, the scaffold-free printing technique could produce the tissue model with improved functionality by providing cells with an environment that allows maintenance of a high intercellular interaction (Kizawa et al., 2017). However, scalability and the heterogeneous structure are limited in scaffold-free printing techniques (Moldovan, 2018). Moreover, several strategies have been studied to produce physiologically relevant 3D *in vitro* liver tissues. In particular, 3D printing technology can effectively simulate the complexity of *in vivo* microenvironments by precisely delivering various types of cells and ECM components. Moreover, as it is flexible in the fabrication process, 3D bioprinting techniques can fabricate multi-scaled or complex heterogeneous constructs, making it applicable for a wide range of liver tissue engineering applications. Using 3D bioprinting techniques, different types of 3D liver tissue models have been designed to emulate the microarchitectural and biological characteristics of native liver tissues for developing physiologically relevant liver tissues (Ma et al., 2016; Grix et al., 2018; Kang et al., 2018; Cui et al., 2019; Mazzocchi et al., 2019; Mao et al., 2020b; Kang et al., 2020).

Hence, in this review, we introduce multicomponent hydrogel bioinks for the 3D bioprinting of liver tissues based on the definition of the bioink and biomaterials that contain cells in it (Groll et al., 2019). We first introduce the 3D printing methods generally utilized for the fabrication of liver tissues—inkjet-based, light-assisted, and extrusion-based 3D printing—and describe the effective factors that should be considered for printing (such as printability and biological performance). Subsequently, multicomponent hydrogel bioinks based on natural biomaterials, which are extensively utilized for bioinks to fabricate liver tissues, are introduced. Next, based on material properties and intrinsic characteristics, we categorize and introduce recent studies for evaluations involving alginate-, collagen-, gelatin-, and decellularized ECM (dECM)-based multicomponent bioinks.. Finally, we introduce the most recent studies on the use of multicomponent bioinks for liver tissue applications with different subjects, such as drug screening, disease models, and liver regeneration models. We believe that this review article can serve as an attractive reference guide for readers who aim to utilize multicomponent bioinks for liver tissue engineering.

## 2 Overview of 3D Bioprinting

Compared with the conventional manufacturing process for tissue engineering, 3D bioprinting enables the fabrication of tissue-mimetic constructs with the desired shapes and biofunctionalities (Kyle et al., 2017; Mao et al., 2020a; Wu et al., 2020). This technique enables the precise distribution of cell-laden bioinks in a layer-by-layer manner in the predefined design; thus, the printed structure can involve cell–cell and cell–matrix interactions, which are not encountered in 2D culture systems (Seol et al., 2014; Mao et al., 2020a; Wu et al., 2020). Additionally, the high process flexibility of 3D bioprinting affords advantages in fabricating multiscale tissue structures with various designs (Kolesky et al., 2016; Gao et al., 2017; Skylar-Scott et al., 2019). In the subsequent subsections, the commonly used 3D bioprinting techniques for liver tissue engineering are introduced briefly.

### 2.1 Types of Bioprinting Techniques: Strengths and Limitations

#### 2.1.1 Inkjet-Based Bioprinting

**FIGURE 1:**
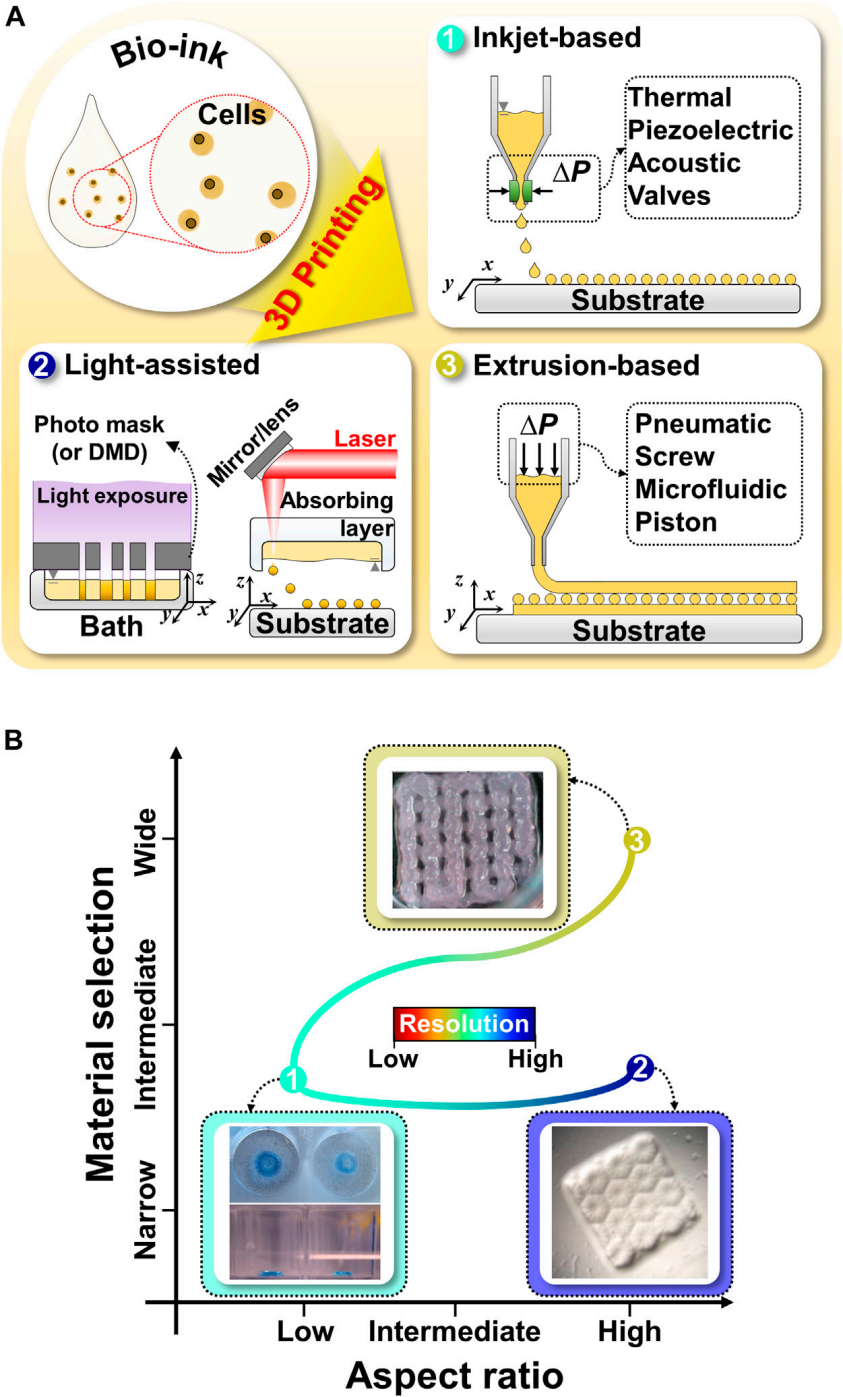
Schematic illustration and comparison of commonly utilized 3D bioprinting techniques for liver tissue engineering **(A)** Schematic illustration of 3D bioprinting techniques, such as inkjet-based bioprinting, light-assisted printing system, and extrusion-based bioprinting. **(B)** Comparison of 3D bioprinting techniques in terms of material selection, aspect ratio, and resolution. Representative images of inkjet-based, light-assisted, and extrusion-based printing. Reproduced with permission from Faulkner-Jones et al. (2015), Ma et al. (2016), Hiller et al. (2018).

Inkjet-based bioprinting utilizes different droplet formation mechanisms; this involves the use of thermal, piezoelectric, acoustic, electromagnetic, or electrohydrodynamic forces to print tissues by successively dropping single droplets of the bioink onto a substrate (Figure 1A) (Matsusaki et al., 2013; Cidonio et al., 2019; Ma et al., 2020). Inkjet-based bioprinting can rapidly fabricate high-resolution structures as it modulates the droplet size more precisely than the filaments generated by extrusion-based bioprinting (Donderwinkel et al., 2017; Ma et al., 2020; Agarwal et al., 2021). Drops with diameters less than 50 μm have also been synthesized (Matsusaki et al., 2013). Additionally, inkjet-based bioprinting is as versatile as extrusion-based bioprinting. Multiple nozzles can be used with inkjet-based bioprinting to print various biomaterials simultaneously for the development of advanced *in vitro* tissue models (Gu et al., 2020). However, the applications of inkjet-based printing are limited because of the required conditions and its drawbacks (Figure 1B). Owing to the principle of inkjet-based bioprinting, the use of low-viscosity bioinks is inevitable. However, such low-viscosity bioinks affect the stability of the printed structures and the formation of new tissues (Gu et al., 2020; Ma et al., 2020). Moreover, inkjet-based bioprinting is associated with problems that can cause the formation of nonuniform droplets, nozzle clogging, and physical stimuli on cells (such as thermal or mechanical stresses) (Gu et al., 2020; Sarkar et al., 2021).

#### 2.1.2 Light-Assisted Bioprinting

Light-assisted bioprinting induces the photopolymerization of a photosensitive polymer by controlling and projecting a light source according to the pattern directed by computer-aided design models (Zhu et al., 2016). Light-assisted bioprinting involves two printing systems: digital light processing (DLP)- and laser-based printing systems. These systems can fabricate structures with higher resolutions than those achievable *via* inkjet- or extrusion-based printing (Donderwinkel et al., 2017; Sun et al., 2018). In particular, DLP-based printing can simultaneously print an entire plane of optical patterns through the selective photocrosslinking of light-activated bioinks according to the light patterns (Figure 1A). In this manner, it significantly reduces the processing time, as compared with the serial printing process of inkjet- and extrusion-based printing (Ma et al., 2016, 2018). Moreover, 3D structures can be manufactured without any intervals generated by basic building units, which makes it possible to fabricate structures with high integrity by using DLP-based printing (Sun et al., 2018; Yu et al., 2019). For example, a research team demonstrated the fabrication of a high-resolution hierarchical vascular network consisting of various widths ranging from 30 to 180 μm in mere seconds using DLP-based printing (Yu et al., 2019). However, only photosensitive polymers can be utilized in light-assisted bioprinting, which narrows the selection of biomaterials and necessitates additional chemical modifications to make them photocrosslinkable (Figure 1B) (Zhu et al., 2016). Furthermore, the byproducts of photocrosslinking mechanisms, light sources, and photoinitiators can cause cell toxicity (Xu et al., 2015; Yue et al., 2015; Donderwinkel et al., 2017; Sun et al., 2018; Mao et al., 2020b).

#### 2.1.3 Extrusion-Based Bioprinting

Unlike inkjet-based printing systems, in extrusion-based printing systems, bioinks in the form of a filament are subjected to a layer-by-layer process to produce 3D structures (Jang, 2017; Sarkar et al., 2021). A continuous filament is extruded from a nozzle by using the force generated by pneumatic pressure or mechanical tools (piston and screw) (Figure 1A) (Jang et al., 2016b). Typically, the resolution of extrusion-based printing is lower than those from the other printing systems, as it is mainly determined by the nozzle’s gauge and properties of the bioinks (Donderwinkel et al., 2017). However, this approach shows good potential for producing 3D tissue models similar to natural tissues (Jang et al., 2018; Gao et al., 2019). First, there is a broader selection of bioinks available than those for inkjet-based and light-assisted printing, including bioinks with high viscosity, high cell density, or various crosslinking mechanisms (Figure 1B) (Kang et al., 2020). These choices provide better options to tune bioink properties to emulate the specific microenvironments of targeted organs (such as biophysical cues, biochemical cues, and cell density) (Kilian et al., 2017). Additionally, a multihead system or co-axial nozzle system can be easily integrated with extrusion-based bioprinting to fabricate 3D heterogeneous tissue-like constructs (Kang et al., 2020; Taymour et al., 2021). For example, a research team created 3D *in vitro* liver tissue model with a structure similar to a liver lobule with a lumen where cells are compartmentalized using a pre-set cartridge (Kang et al., 2020). Moreover, it can provide imparted macropores and vessel-like perfusable channels to a 3D volumetric printed structure to supply oxygen and nutrients to the embedded cells using printing systems with a sacrificial material (Jia et al., 2016; Kolesky et al., 2016; Kilian et al., 2017; Taymour et al., 2021). Thus, the versatility of extrusion-based printing has introduced a new avenue for the production of biomimetic tissues and organs at the human clinical scale (Jang et al., 2018).

## 3 Overview of Bioink Design Considerations for 3D Bioprinting

As interest in biofabrication increases, the definition of bioink has been varied. In this review article, we defined the bioink as cell-encapsulated biomaterial that enables cells to be cultivated stably *in vitro* (Malda et al., 2013; Gao et al., 2017; Jang et al., 2017; Groll et al., 2019; Kang et al., 2020). Bioink is the main component of 3D bioprinting affecting the printing process and functions of 3D-printed tissues. Bioink viscosity can determine 1) the shape retention of the bioink deposited during the printing process and 2) the finishing accuracy of the printed structure (Chimene et al., 2016). Additionally, the crosslink density of the bioink can adjust the post-printing mechanical properties, which affects the long-term maintenance of shape fidelity and structural stability (Kyle et al., 2017). Conversely, the biochemical and biophysical characteristics of the bioink facilitate the cellular growth and motility of cells embedded in the printed structure and also guide tissue formation (Park et al., 2016; Kilian et al., 2017). In the following subsections, we discuss certain important considerations pertaining to the preparation of optimal bioinks.

### 3.1 Key Bioink Considerations

#### 3.1.1 Printability

Bioinks need to exhibit excellent shape fidelity during printing and maintain their initial 3D shape after printing because the printability of the bioink determines the size and structure accuracy of the 3D-printed construct (Jang et al., 2018; Mao et al., 2020a). Appropriate extrudability, filament formation, and shape fidelity are all considered for printability evaluation (Schwab et al., 2020). The rheological properties and crosslinking mechanisms of bioinks are related to printability. Viscosity is a rheological property and refers to the generated fluid resistance when the applied force causes a flow (Malda et al., 2013; Cui et al., 2020). Because high viscosity can prevent the collapse of printed structures, high-viscosity bioinks are preferred in 3D printing, as they help improve printability. The viscosity of a bioink is mainly related to the concentration of the polymers and the molecular weight (Cui et al., 2020). Printing parameters such as the temperature and shear rate can also help adjust the viscosity of bioinks. It is, therefore, necessary to precisely tune the viscosity of bioinks in order to fabricate the desired structures with high shape fidelity.

Crosslinking and gelation play critical roles in stabilizing the printed construct for maintaining shape fidelity over prolonged time periods. Crosslinking is commonly classified into physical and chemical subtypes. Physical crosslinking involves the formation of a physical network via various noncovalent interactions between polymeric chains, such as hydrogen bonding and hydrophobic, electrostatic, and guest–host interactions (Akhtar et al., 2016; Li and Lin, 2021). Conversely, the chemical crosslinking process depends on various reaction mechanisms, inducing covalent bonds between the reactive functional groups (Parhi, 2017; Cui et al., 2020). Crosslinking methods are typically employed during printing or post-printing to produce structures with high shape fidelity. Hence, it is necessary to understand the physical and chemical methods for crosslinking hydrogels that can be applied to the development of bioinks with high printability (Cui et al., 2020).

#### 3.1.2 Biological Performance

Bioinks should exhibit biochemical and biophysical properties similar to those of the ECM of the targeted tissue for cell growth and proliferation (Benwood et al., 2021). The biological performance of bioinks influences cell viability, functions, behavior, and cell-mediated matrix modeling (Gao et al., 2019). Biochemical and biophysical interactions between cells and the surrounding matrix stimulate the critical signal process related to the mediation and direction of cellular development (Pati et al., 2014). Consequently, the biochemical and biophysical characteristics of the ECM can dictate the cell fate, affect cell morphology, and induce tissue-specific differentiation (Muncine and Weaver, 2018). With regard to improving the biochemical properties of bioinks, the introduction of additional bioactive components to the bioinks facilitates cell adhesion and enhances cell functions and proliferation (Heid and Boccaccini, 2020). Furthermore, it can guide cellular behavior by modifying the biophysical properties, such as stiffness of the matrix, according to the application purpose. For instance, based on the matrix-stiffness-mediated stem cell (SC) differentiation direction, osteogenic lineage differentiation occurs in a stiffer matrix, whereas myogenic lineage differentiation occurs in a softer matrix (Engler et al., 2006; Guilak et al., 2009). As a result, bioinks need to satisfy both biochemical and biophysical properties for *in vivo*-like tissue development.

### 3.2 Limitations of Single-Component Bioinks

An ideal bioink needs to possess biochemical and biophysical (such as mechanical, chemical, and biological) properties similar to those of natural tissues and high printability characteristics (Ahadian and Khademhosseini, 2018). However, single-component bioinks cannot satisfy both these critical requirements for the successful bioprinting of functional tissue constructs (Gopinathan and Noh, 2018; Cui et al., 2020). As these requirements constrain each other, a single-component bioink can only possess either printability or functionality, thus resulting in a narrow range of printable tissue structures (Gu et al., 2020). For example, high viscosity or high crosslinking density is required to maintain shape fidelity and prevent the printed construct from collapsing; however, this forms a stiff microenvironment that limits cellular behavior (Bell and Haycock, 2012; Kyle et al., 2017). By contrast, a soft matrix is favorable for cells to proliferate and migrate, although this causes difficulties in maintaining structural stability (Kyle et al., 2017). Additionally, even though existing natural biomaterials [such as collagen, alginate, hyaluronic acid (HA), fibrin, and gelatin] are biocompatible for cell growth and proliferation, the intrinsic morphology and function of living tissues cannot be achieved using these biomaterials, as they fail to emulate the complexity of the natural ECM (Sellaro et al., 2010; Pati et al., 2014). Therefore, the development of printable bioinks that afford a tissue-specific microenvironment to expand the biofabrication window is important.

## 4 Multicomponent Hydrogel Bioinks for 3D Bioprinting of Liver Tissues

Multicomponent bioinks are described as a combination of two or more biomaterials. In this manner, they can reinforce the limited printability and biological performance of single-component bioinks (Ashammakhi et al., 2019; Cui et al., 2020). For example, in the case of multicomponent bioinks comprising different polymers, the rheological properties of the bioinks can be adjusted during and after printing in order to achieve high printability and structural stability. Furthermore, bioactive or extracellular components can be added to improve the biological performance of single-component bioinks (Cui et al., 2020). Therefore, it is desirable to further develop bioinks to provide a more *in vivo*-like microenvironment, while ensuring high printability (Choi et al., 2021). For bioprinting liver tissue models, multicomponent bioinks consisting of natural biomaterials, including semisynthetic materials [such as gelatin methacrylate (GelMA)], have been utilized to replicate the properties of the liver tissue matrix closely, except for synthetic biomaterials. Hence, we discuss the multicomponent bioinks based on natural biomaterials with a molecular structure similar to that of ECM components performing structural functions, which are mainly employed in 3D bioprinting liver tissues.

### 4.1 Types of Multicomponent Bioinks

**FIGURE 2:**
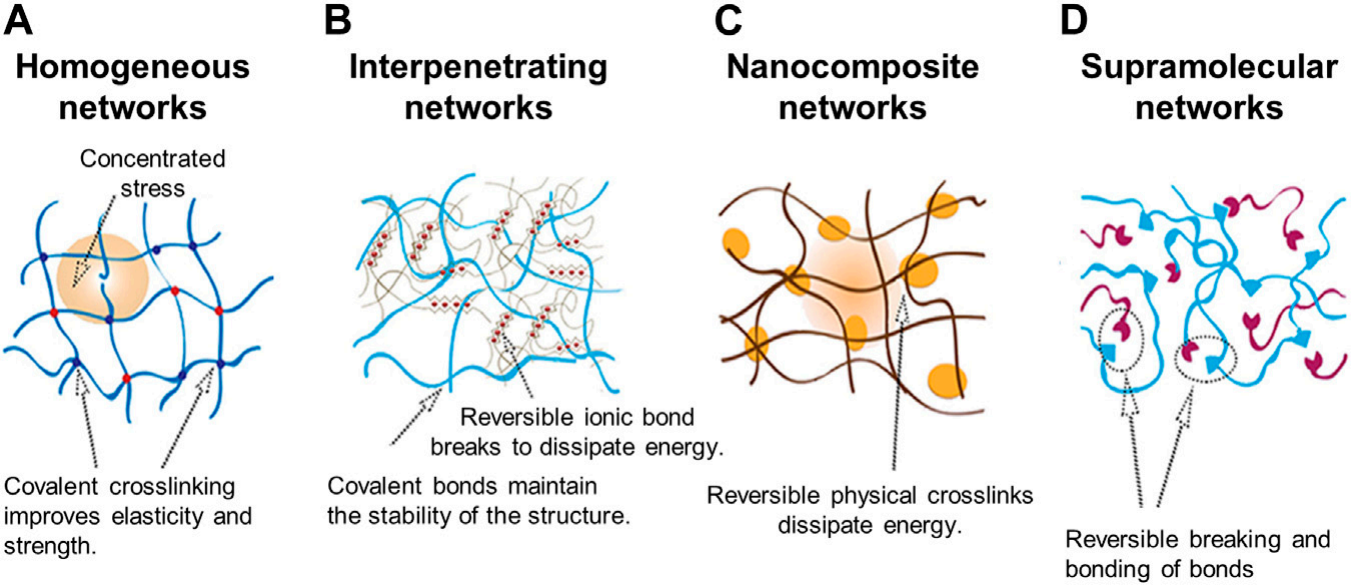
Schematic illustration of four interconnecting networks in multicomponent hydrogel bioinks **(A)** Homogeneous networks **(B)** Interpenetrating networks **(C)** Nanocomposite networks **(D)** Supramolecular networks. Reproduced with permission from (Li et al., 2021).

Typically, multicomponent bioinks are classified into four categories (Chimene et al., 2020; Cui et al., 2020). Multimaterial bioinks contain two or more types of biomaterials that can be covalently crosslinked with each other (Figure 2A) (Chimene et al., 2016; Ashammakhi et al., 2019). Conversely, interpenetrating network bioinks include biomaterials that form individual networks based on independent crosslinking mechanisms, whereby the networks are intertwisted without covalent bonds between the networks in the printed construct (Figure 2B). As a result, the mechanical stability of the printed structure can be improved by increasing the number of polymers involved in the formation of the crosslinking networks in the resultant hydrogel (Cui et al., 2020). Moreover, interpenetrating network bioinks can be used to print structures with high fidelity by inducing the crosslinking of the biomaterials during the printing process. For example, during printing, the photocrosslinking of GelMA or the ionic crosslinking of alginate occurs first to preserve the printed shape. In addition, the shape stability of the construct can be further improved by the secondary crosslinking process of the remaining materials (Colosi et al., 2016). Nanocomposite bioinks include nanoparticles or nanostructures that can increase the stiffness of the printed structure and enhance the shear-thinning behavior (Figure 2C). Additionally, nanocomposites can affect cellular behavior by increasing bioactivity. In particular, nanocomposite bioinks containing conductive nanobiomaterials are used to fabricate tissues that require electric signals, such as muscles and nerves. Supramolecular bioinks adopt the supramolecular crosslinking method via a guest–host bond between the functional groups attached to a biomaterial, forming and breaking the reversible bonds according to the developed stress (Figure 2D) (Jang et al., 2018). This self-healing ability not only causes shear-thinning behavior but also enables the formation of a network without any chemical reagents and physical stimuli that can potentially affect cell viability.

### 4.2 Multicomponent Hydrogel Bioinks Based on Natural Biomaterials

#### 4.2.1 Chitosan-Based Multicomponent Bioinks

Chitosan is a natural biomaterial obtained by the deacetylation of chitin, and it exhibits good biocompatibility, biodegradability, and antibacterial properties. In addition, it has a molecular structure similar to that of glycosaminoglycan, a representative component of the ECM (Jiankang et al., 2009). Moreover, as chitosan can be readily processed, it has been used extensively in tissue engineering in various forms, including gels, membranes, nanofibers, beads, nanoparticles, porous scaffolds, and sponges. Chitosan has also been used extensively in liver tissue engineering. It was reported that several liver-specific functions are improved when the hepatocytes are cultured on porous chitosan scaffolds *in vitro* (Li et al., 2003; Li et al., 2004; Jiankang et al., 2009; Rajendran et al., 2017). However, chitosan is insoluble in water under physiological conditions, and its sediments are formed when the pH is higher than 6.2; this makes it unsuitable for use as a bioink composed of cells (Mora Boza et al., 2019). As a result, most chitosan-based biomaterials used in liver tissue engineering were adopted to fabricate porous acellular scaffolds (Jiankang et al., 2009; Tripathi and Melo, 2015; Rajendran et al., 2017). However, recently, various studies have attempted to make chitosan cross-linkable under physiological conditions. Nevertheless, a 3D-bioprinted liver structure with chitosan-based multicomponent bioinks has not been reported thus far.

#### 4.2.2 Alginate-Based Multicomponent Bioinks

Alginate hydrogel is one of the most extensively used biomaterials in tissue engineering, such as for the heart, bone, cartilage, and liver (Jang et al., 2018; Ye et al., 2019; Sarkar et al., 2021). Alginate is a biopolymer found in brown seaweed or algae (Duin et al., 2019; Ye et al., 2019; Piras and Smith, 2020; Sarkar et al., 2021). As alginate is a negatively charged polysaccharide, alginate hydrogel can be readily polymerized by reacting with multivalent cations, such as $Ca^{2+},\;Sr^{2+},\;\;\text{and}\;Ba^{2+}$ (Lee et al., 2000; Kong et al., 2002; Jang et al., 2018; Duin et al., 2019; Ye et al., 2019; Piras and Smith, 2020; Wu et al., 2020; Sarkar et al., 2021). Moreover, alginate is biocompatible and controllable in terms of its mechanical properties and printability, thus making it applicable in several bioprinting techniques (Suntornnond et al., 2017; Tai et al., 2019; Davoodi et al., 2020; Piras and Smith, 2020) and for fabricating various forms of tissue constructs (Huang et al., 2012; Onoe et al., 2013; Andersen et al., 2015; Piras and Smith, 2020). However, it is difficult for cells to adhere because of the bioinert nature of the biomaterial (Gao et al., 2017; Sarkar et al., 2021), and alginates at cytocompatible concentrations have low viscosity, thus making printing difficult.

**FIGURE 3:**
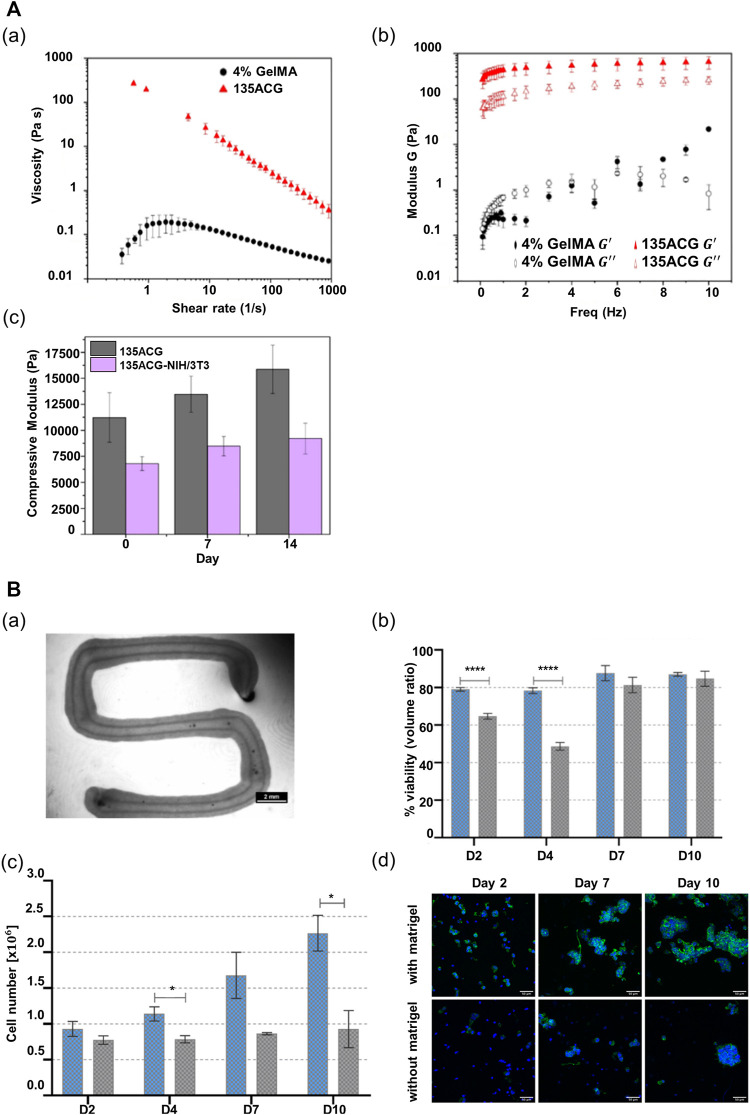
Alginate-based multicomponent bioinks. **(A)** Rheological property of two different bioinks, 4% GelMA and 135ACG. (a) Flow behaviors of two different bioinks. (b) Elastic modulus ( $G^{'}$ ) and viscous modulus ( $G^{"}$ ) of the two different bioinks as a function of oscillatory frequency. (c) Compressive modulus changes of 135ACG gels with/without cells over 14 days. Reproduced with permission from Wu et al. (2020). **(B)** Characteristics of algMC bioink. (a) Stereo microscopic image of a cell-free core–shell strand with compartmentalized core and shell structure within the strand. Scale bar: 2 mm. (b) Viability of HepG2 cells encapsulated in algMC bioink comparing two conditions (with matrigel vs without matrigel) (*n* = 6, mean ± SD, ****p < 0.0001). (c) Proliferation of HepG2 cells encapsulated in algMC bioink comparing two conditions (with matrigel vs without matrigel) (*n* = 3, mean ± SD, *p < 0.05). (d) Morphology and organization of HepG2 encapsulated in algMC comparing two conditions (with matrigel vs without matrigel). Reproduced with permission from Taymour et al. (2021).

To improve the performance of alginate, it is necessary to combine it with other materials that can alter its properties, including nanomaterials that can change its rheological properties and a peptide motif that can promote cell adhesion (Piras and Smith, 2020; Sarkar et al., 2021). Wu et al. (2020) incorporated cellulose nanocrystals and GelMA in alginate to improve its printability and biological performance. The alginate-based multicomponent bioink (ACG) showed strong shear-thinning behavior suitable for extrusion-based printing, despite the low-alginate concentration (1% w/v) in the composite bioink (Figure 3Aa). Additionally, the elastic modulus of the ACG bioink showed a considerable value, as compared with the viscous modulus in all frequency ranges up to 10 Hz; this demonstrates that the shape of the printed structure can be supported in a stable manner after printing (Figure 3Ab). Moreover, the photopolymerization of GelMA and the electrostatic interaction of alginate result in an independent interpenetrating network, maintaining high structural stability after printing. In addition, unlike pure alginate, the composite bioink afforded appropriate stiffness and cell-binding motifs to promote cell growth and cellular behavior, including cell adhesion, spreading, and proliferation (Figure 3Ac). The compressive modulus of the composite bioink exceeded 10 kPa, which facilitates the behavior of supporting cells NIH/3T3 that influence hepatocyte functions (Figure 3A).

In another research study, Taymour et al. (2021) combined methylcellulose with alginate to improve the printing properties of alginate. They showed that a mixture of alginate and methylcellulose (algMC) can provide a cytocompatible environment and also be employed for the fabrication of volumetric structures with high shape fidelity (Schütz et al., 2017; Duin et al., 2019; Ahlfeld et al., 2020). In this previous study, they employed the algMC bioink in an extrusion-based printing system with a co-axial nozzle. Both the core and the shell contained this algMC bioink, and as a result, they fabricated a core–shell compartmentalized strand (Figure 3Ba). Interestingly, it was shown that the structure maintained shape stability without any additional crosslinking agent. Moreover, the researchers added matrigel to the algMC bioink to provide a physiologically relevant microenvironment for hepatocytes. The viability and proliferation of hepatocytes improved in the algMC bioink with the addition of matrigel (Figures 3Bb,c). Additionally, in the algMC supplemented by matrigel, HepG2 formed a giant cluster with a prominent actin structure and a higher number of cell clusters, as compared with the HepG2 cultured in matrigel-free algMC (Figure 3Bd).

#### 4.2.3 Collagen-Based Multicomponent Bioinks

Collagen is broadly employed for bioprinting as an ideal biomaterial to provide cells with an *in vivo-*like microenvironment (Mazzocchi et al., 2019; Nguyen et al., 2019). As it is a significant component of the ECM, collagen can structurally support embedded cells and biochemically regulate cell functions and behaviors (such as metabolism, proliferation, cell adhesion, and cell migration) (Ye et al., 2019; Cui et al., 2020; Patil and Masters, 2020; Benwood et al., 2021). In addition, it was reported that collagen type I can regulate liver cell phenotypes (Wang et al., 2016). Moreover, it is preferred in 3D printing applications as it can increase the structural stability by inducing thermal crosslinking within a physiological temperature range (∼37°C) (Jang et al., 2016b; Gopinathan and Noh, 2018; Benwood et al., 2021). However, hydrogels that are only composed of collagen are inappropriate for 3D bioprinting owing to their low mechanical properties and uncontrolled gelation characteristics (Gopinathan and Noh, 2018; Mazzocchi et al., 2019; Benwood et al., 2021). Despite the limited printability of collagen, the inherent biological performance of collagen has led to the development of various collagen-based bioinks (Gopinathan and Noh, 2018; Mazzocchi et al., 2019; Patil and Masters, 2020).

**FIGURE 4:**
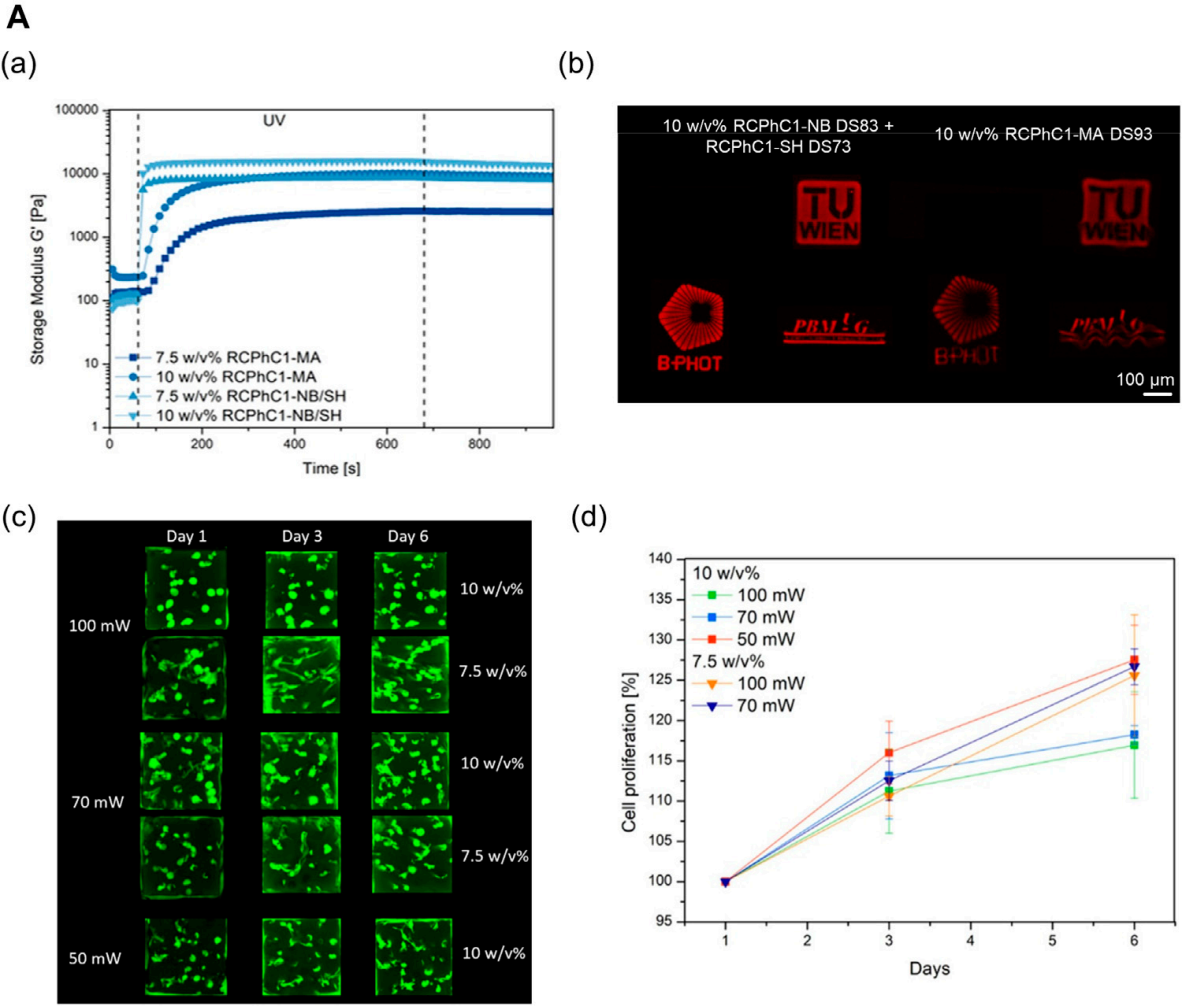
Collagen-based multicomponent bioinks. **(A)** Characteristics of photocrosslinkable modified collagen bioink (Methacrylamide-modified RCPhC1 (RCPhC1-MA), Norbornene-functionalized RCPhC1 (RCPhC1-NB), and Thiolated RCPhC1 (RCPhC1-SH)) (a) Rheological property on 7.5 and 10 w/v % solutions of RCPhC1-NB/SH and RCPhC1-MA in the presence of 2 mol% Li-TPO-L at 37°C. (b) Printability test of photocrosslinkable collagen-based bioinks using different logos (TU Wien, B-PHOT, and PBM) (left panel – 10 w/v % RCPh1-NB/SH, right panel – RCPhC1-MA). Scale bar: 100 µm. DS: degree of substitution. (c) Laser scanning microscopy (LSM) images of living ASCs-GFP printed in RCPhC1-NB/SH-based cubes at different polymer concentrations and laser power intensities. (d) Cell proliferation trends as a function of time in cubes printed using RCPhC1-NB/SH at different polymer concentrations and laser power conditions. Reproduced with permission from (Tytgat et al., 2020).

Efforts have been devoted toward designing collagen-based multicomponent bioinks to provide a more physiologically relevant targeted tissue environment while ensuring printability by mixing naturally derived biopolymers (Patil and Masters, 2020). For example, a combination of biomaterials presents more stable rheological properties than pure collagen. Thus, biomaterial blending can help manufacture complex constructs using the hybrid bioink without requiring supporting materials or chemical crosslinkers (Duarte Campos et al., 2019). Furthermore, modified collagen bioinks can be photocrosslinked rapidly to develop biocompatible bioinks with high printability characteristics (Figure 4A) (Tytgat et al., 2020). In addition, in the domain of liver tissue engineering, Mazzocchi et al. (2019) determined the optimal ratio of methacrylated collagen I and thiolated HA to improve printability and bioactivity. The collagen-based multicomponent hydrogel they developed showed improved elastic properties, as compared with standard HA/gelatin hydrogels. Moreover, this hydrogel type can be strengthened under ultraviolet (UV) light conditions with two independent crosslinking systems: the photopolymerization of methacrylated collagen I and the thiol-ene reaction of thiolated HA. Thus, the high fidelity of the structure can be realized after printing. In terms of bioactivity, the high cell viability and *in vivo*-like morphology of the cells were preserved more effectively in the collagen-based multicomponent bioink.

#### 4.2.4 Gelatin-Based Multicomponent Bioinks

Gelatin, a collagen derivative, has been extensively used in 3D printing applications as an attractive alternative to collagen (Mao et al., 2020a). As gelatin closely shares collagen’s molecular structure and function, gelatin can be used for an ECM-like microenvironment to cells (Jang et al., 2016b; Bello et al., 2020). In particular, the gelatin-rich arginine-glycine-aspartic acid (RGD) motif used for cell attachment can promote certain cell behaviors (such as cell adhesion, migration, and proliferation) (Cui et al., 2019; Ye et al., 2019; Bello et al., 2020; Sarkar et al., 2021). In addition, unlike collagen, gelatin can reversibly form a collagen-like intermolecular network based on a physical gelation process (Lee and Mooney, 2001; Sarkar et al., 2021). Consequently, it has been more widely used for tissue printing than collagen. For example, given that the sol–gel transition of gelatin can occur reversibly in the physiological temperature range, gelatin can be used as a fugitive material for bioprinting (Jang et al., 2016b, 2018). However, the use of pure gelatin for bioprinting is challenging because of its poor thermal stability and lack of mechanical properties at low viscosities (Bello et al., 2020; Cui et al., 2020; Wu et al., 2020). In particular, the tendency of gelatin to liquefy at high temperatures (∼37°C) substantially impedes its application in the development of bioink (Wu et al., 2020). Furthermore, gelatin suffers from limitations as a biomaterial for *in vitro* tissue development because it does not provide any bioactive factors, except for cell-binding motifs (Somasekharan et al., 2020). Hence, to enhance the printability and bioactivity of gelatin, gelatin-based bioinks containing various biomaterials have been developed and employed for bioprinting (Jang et al., 2018; Ashammakhi et al., 2019; Bello et al., 2020).

**FIGURE 5:**
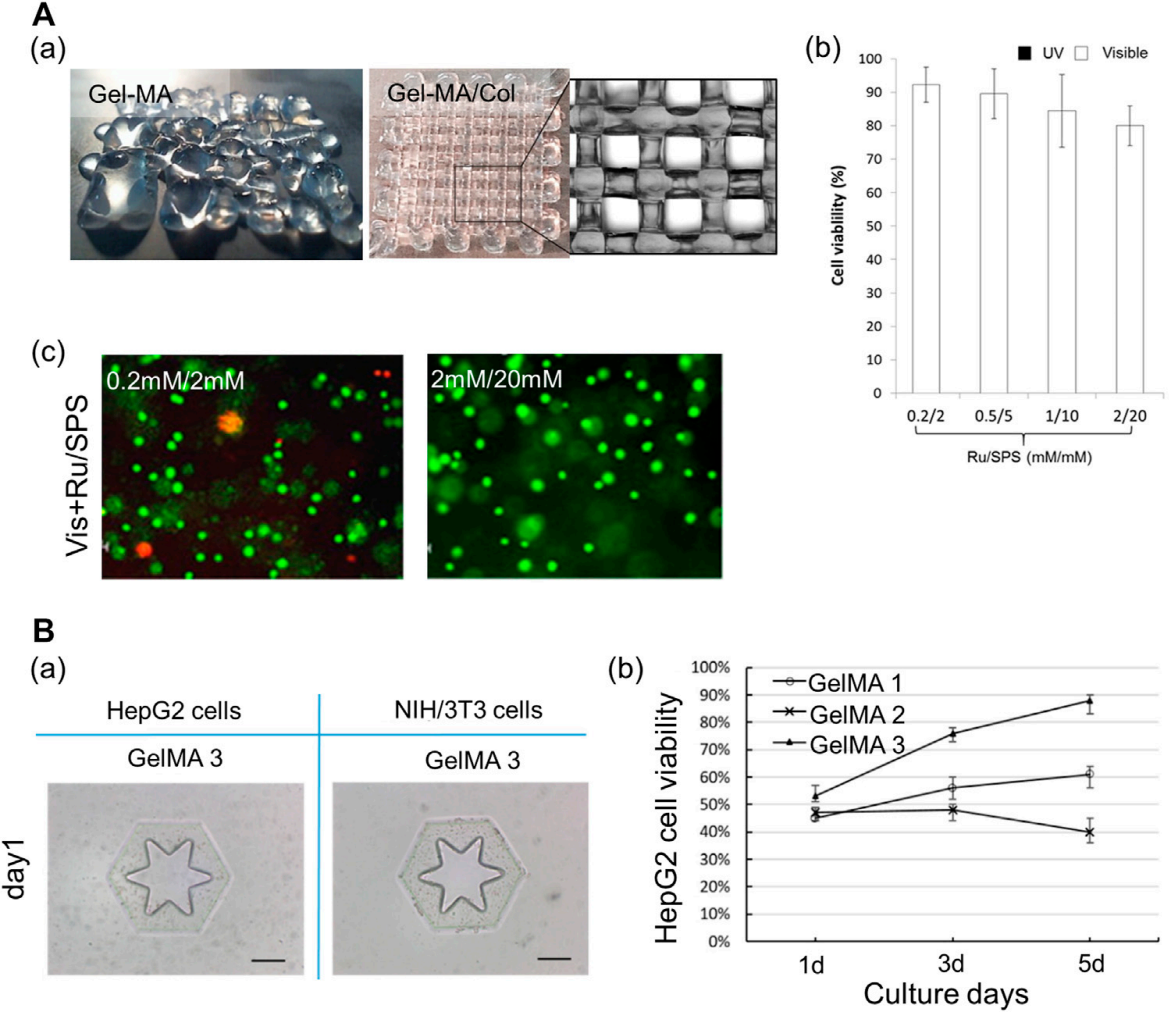
Gelatin-based multicomponent bioinks. **(A)** Characteristics of hybrid bioink of GelMA and collagen. (a) Shape fidelity of the acellular construct fabricated by GelMA and hybrid bioink of GelMA and collagen. Scale bar: 1 mm. (b) Cell viability of the MCF-7 encapsulated in GelMA/collagen with different Ru/SPS concentrations. (c) Live dead images of MCF-7 encapsulated in the hybrid bioink of GelMA and collagen with photoinitiator Ru/SPS (0.2 mM/2 mM and 2 mM/20 mM). Scale bar = 100 μm. Reproduced with permission from Lim et al. (2016). **(B)** Characteristics of GelMA. (a) Shape fidelity of the cell-laden construct fabricated by GelMA 3. Scale bar: 200 μm. (b) The viability of HepG2 cells respective encapsulated in GelMA 1, GelMA 2, and GelMA 3 during 5 days culture. Reproduced with permission from Cui et al. (2019).

For printability, gelatin is directly conjugated with a photopolymerizable functional group (Jang et al., 2016b; Cui et al., 2020; Sarkar et al., 2021) or mixed with other hydrogels according to their distinct crosslinking mechanisms (Gao et al., 2017). For example, the mixture of alginate and gelatin can improve the printability of gelatin via the rapid ionic crosslinking of alginate (Xie et al., 2021). Furthermore, matrigel or the ECM can be employed to enhance the bioactivity of gelatin-based bioinks (Hiller et al., 2018; Mao et al., 2020c). The printed structure using GelMA with a collagen blend represented higher shape fidelity than that with pure GelMA. Moreover, the photocrosslinkable hydrogel with ruthenium (Ru)/sodium persulfate (SPS), which can react under visible light conditions, can provide a more cytocompatible environment than those of the other photoinitiators (Figure 5A) (Lim et al., 2016). Furthermore, ECM components are added to the gelatin hydrogel to strengthen bioactivity (Somasekharan et al., 2020). Cui et al. (2019) determined the optimal synthetic conditions for gelatin and methacrylic anhydride suitable for DLP-based 3D bioprinting techniques. Three types of GelMA were synthesized by varying the concentration of methacrylic anhydride, and these were compared. GelMA with the highest concentration of methacrylic anhydride (20% w/v) underwent crosslinking rapidly (0.5 s), and the structure printed using this bioink exhibited high structural fidelity. Moreover, it maintained a stable structural shape for 10 days. Furthermore, it has been demonstrated that this bioink can provide an appropriate microenvironment for cells. Based on the live/dead cell fluorescent images, high cell proliferation was maintained in this GelMA, as compared with that in the GelMA with a low degree of methacrylic anhydride (Figure 5B). Ma et al. (2016) applied the GelMA with stiffness comparable to that of healthy liver tissues to support the hepatocytes in maintaining cell functions and promoting cellular behaviors, including cell migration and proliferation. Furthermore, the researchers used a combination of glycidyl methacrylate hyaluronic acid (GMHA) and GelMA to provide a more bioactive microenvironment for the endothelial cells, given that HA facilitates the proliferation of endothelial cells and supports vascularization (Leach et al., 2003; Ma et al., 2016). In addition, as both bioinks exhibited high shape fidelity *via* rapid photocrosslinking, a high-resolution patterned structure with high shape stability could be realized.

#### 4.2.5 dECM-Based Multicomponent Bioinks

dECM hydrogel has recently been considered a promising biomaterial for bioprinting applications. dECM is obtained based on the decellularization process to delete cellular components from native tissues. Physical, chemical, and enzymatic agents, or combinations thereof, are included in decellularization methods. Interestingly, it is clear that the optimal decellularization method may differ depending on the tissue and organ owing to tissue-specific characteristics (such as cell density, matrix density, tissue thickness, and lipid content) (Crapo et al., 2011; Choudhury et al., 2018). Therefore, for the development of dECM that exhibits more *in vivo* relevance and can be applied clinically, various decellularization methods and materials are being studied and developed to effectively remove potential components that can cause immune rejection, while minimizing damage to the ECM structure (Crapo et al., 2011; Keane et al., 2015; Fernández-Pérez and Ahearne, 2019). For example, to produce a dECM bioink, the decellularization method that employs perfusion and minimizes ECM structure damage was used (Sharma et al., 2021).

**FIGURE 6:**
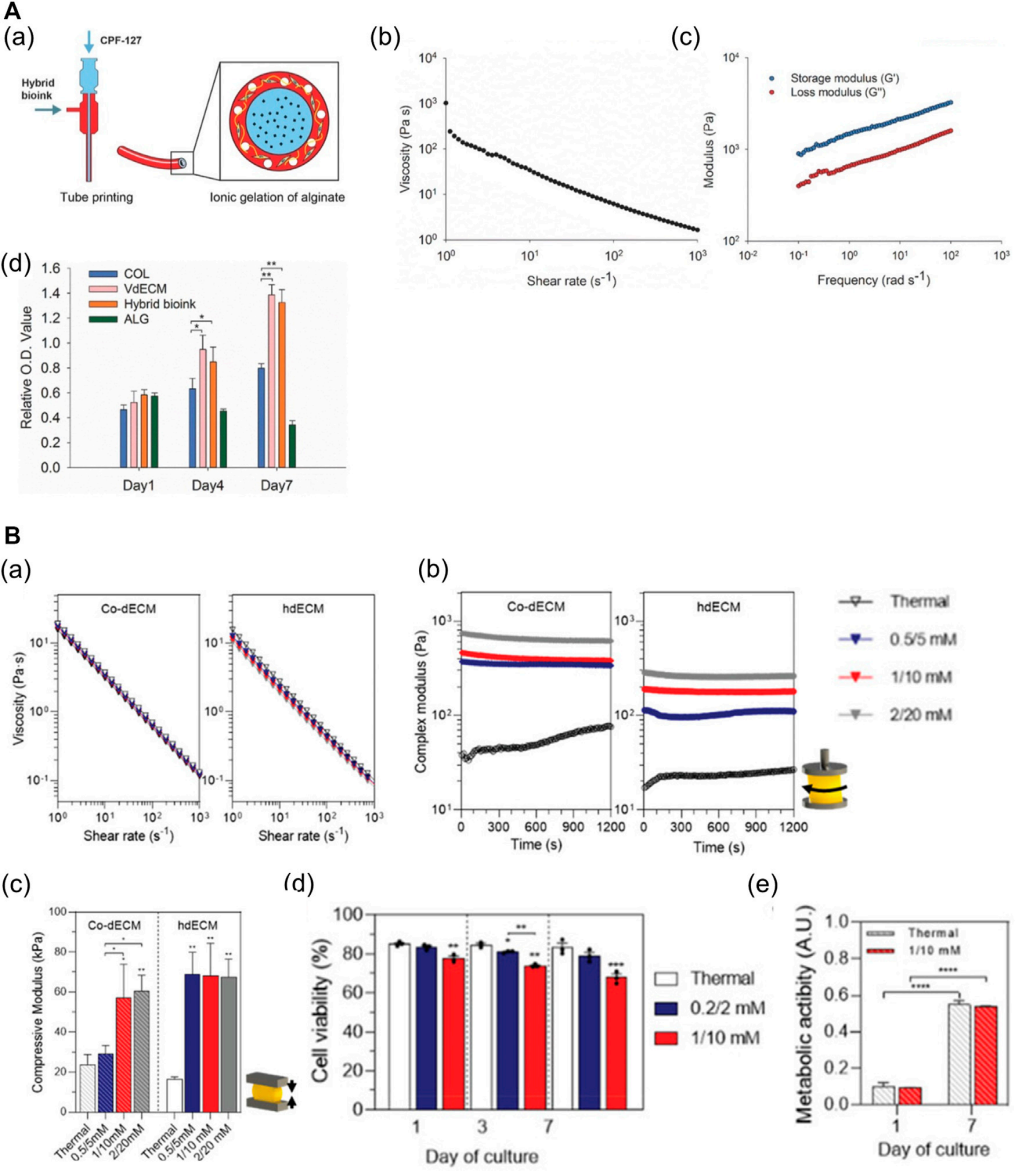
dECM-based multicomponent bioinks. **(A)** Characteristics of hybrid bioink of VdECM and alginate. (a) Schematic depiction of the bio-blood vessel (BBV) printing using hybrid bioink with co-axial nozzle. (b) Shear-thinning behavior of the hybrid hydrogel. (c) The complex modulus of crosslinked hybrid bioink. (d) The proliferation rate of endothelial progenitor cells (EPCs) encapsulated in different types of bioinks (*p < 0.1, **p < 0.01). Reproduced with permission from Gao et al. (2017). **(B)** Characteristics of dECM bioinks with Ru/SPS (dERS). (a) Shear-thinning behavior of two different dECM-based dERS bioinks (cornea dECM (co-dECM) and heart dECM (hdECM)). (b) The complex modulus of crosslinked dERS bioinks compared with dECM bioinks. (c) Improved mechanical properties of dERS bioinks after photocrosslinking and thermal crosslinking process. *p < 0.1, **p < 0.01. (d) Cell viability of hiPSC-CMs embedded in the printed construct with hdECM-based bioinks at days 1, 3, and 7 (hdECM vs hdERS). (e) Metabolic activity of predifferentiated keratocytes in the printed construct with co-dECM bioinks at day 1 and day 7 (co-dECM vs co-dERS). ***p < 0.001. Reproduced with permission from Kim et al. (2021).

As dECM preserves the unique components and structures of ECM that are difficult to emulate with single-component biomaterials, dECM hydrogels can support the cells structurally and orchestrate the cell dynamics and behaviors by providing tissue-specific microenvironments (Pati et al., 2014; Han et al., 2019; Abaci and Guvendiren, 2020; Mao et al., 2020b). Replicating biochemical and biophysical properties comparable with those of the matrix of tissues can improve cell viability and function, and promote tissue development (Pati et al., 2014; Jang et al., 2016a; Das et al., 2019; Kim et al., 2020a; Mao et al., 2020b). In addition, it has been reported that specific organ-derived dECM induces the differentiation of SCs into the organ cell types (Lee et al., 2017b; Han et al., 2019). Moreover, dECM hydrogels abundant in collagenous proteins readily undergo gelation at physiological temperatures, which enables bioprinting. However, owing to the low mechanical stability and slow dECM gelation mechanism, the fabrication of complex or large structures with high shape fidelity is challenging (Abaci and Guvendiren, 2020; Mao et al., 2020b; Sarkar et al., 2021). Hence, approaches to improve the printability of dECM bioinks have been developed, such as blending with other cross-linkable hydrogels (Gao et al., 2017; Ma et al., 2018; Mao et al., 2020b), direct combination with the methacrylate group (Kim et al., 2020b), and addition of photocrosslinkers (Jang et al., 2016a; Abaci and Guvendiren, 2020; Kim et al., 2021). For example, a mixture of the vascular-tissue-derived decellularized extracellular matrix (VdECM) and alginate enabled precise printing via rapid ionic alginate crosslinking and supported the stability of the structure via thermal dECM crosslinking (Figure 6A) (Gao et al., 2017). Furthermore, it was demonstrated that the dECM hydrogel mixed with Ru/SPS (dERS) can be photocrosslinked rapidly, and used to fabricate complex structures with dERS without cell damage (Figure 6B) (Kim et al., 2021).

Likewise, in the field of bioprinting liver tissues, Ma et al. (2018) mixed liver dECM and GelMA to develop the photocrosslinkable liver-derived dECM (LdECM)-based bioink for DLP-based 3D bioprinting. As it can be crosslinked rapidly via GelMa photocrosslinking, the printed structure exhibited high fidelity, and the printed shape remained stable for 7 days. In addition, the photocrosslinkable bioink exhibits flexibility in that the mechanical properties can be altered based on the light exposure time. Furthermore, the multicomponent bioink provides a better liver-specific microenvironment than a single ECM component. It maintained high cell viability for 7 days, and the liver-specific marker of the cell encapsulated in the multicomponent bioink was expressed at higher levels than those by a single ECM component. As such, hybrid LdECM (in conjunction with GelMA) has been used to provide targeted tissue-specific microenvironments and concurrently presents high shape fidelity via rapid photocrosslinking (Yu et al., 2019; Mao et al., 2020b). Kim et al. (2020b) determined the optimal ratio between liver dECM and a gelatin mixture for extrusion-based 3D bioprinting. Their research developed LdECM-based multicomponent bioinks by blending liver dECM particles smaller than 100 μm with a gelatin mixture (gelatin, HA, and fibrinogen). They compared three types of multicomponent bioinks with different concentrations of liver dECM. The three liver dECM-based multicomponent bioinks exhibited good rheological properties for extrusion-based 3D bioprinting and a compressive modulus <10 kPa. Moreover, the 2% w/v liver dECM pBio-ink exhibited higher shape fidelity in 2D and 3D patterning than the 2% w/v conventional liver dECM bioink group and gelatin group. Moreover, it maintained high cell viability and enhanced the cell function in this liver dECM-based multicomponent bioink. The liver-specific marker of the liver cells in this multicomponent bioink was expressed similarly for 14 days to that of the cells in liver dECM. Multicomponent bioinks based on different natural biomaterials for 3D bioprinting liver tissues are summarized in Table 1.

**TABLE 1:** Summary of multicomponent bioinks based on different natural biomaterials for 3D bioprinting liver tissue.

Bioink category	Applied materials	Characteristics				Ref.
		Cell viability	Biological activity	Crosslinking rate	Shape fidelity	
Alginate-based	Alginate + cellulose nanocrystal + GelMA	High	High (to moderate)	Rapid	High (to moderate)	Wu et al. (2020)
	Alginate + methylcellulose + matrigel	High	High	Rapid	High (to moderate)	Taymour et al. (2021)
Collagen-based	Methacrylated collagen type I + thiolated hyaluronic acid	High	High (to moderate)	Rapid	High	Mazzocchi et al. (2019)
Gelatin-based	Gelatin + alginate	High	High (to moderate)	Rapid	Moderate	Yang et al. (2021)
	Gelatin + alginate	High	High (to moderate)	Rapid	Moderate	Xie et al. (2021)
	Gelatin + alginate + matrigel	High	High	Rapid	N/A	Mao S. et al. (2020)
	Gelatin + alginate + human lung dECM	High	High	Rapid	High (to moderate)	Hiller et al. (2018)
	GelMA	High (to moderate)	N/A	Rapid	High	Cui et al. (2019)
	GelMA	High	High	Rapid	High	Bhise et al. (2016)
	GelMA + GMHA	High (to moderate)	High (to moderate)	Rapid	High	Ma et al. (2016)
dECM-based	Liver dECM + GelMA	High	High	Rapid	High	Ma et al. (2018)
	Liver dECM + gelatin mixture (gelatin + hyaluronic acid + fibrinogen)	High	High	Moderate	High (to moderate)	Kim W. et al. (2020)
	Liver dECM + GelMA	High	High	Rapid	High	Mao Q. et al. (2020)
	Liver dECM + GelMA	High	High	Rapid	High	Yu et al. (2019)

## 5 3D-Bioprinted Liver Tissue Models With Multicomponent Hydrogel Bioinks Based on Natural Biomaterials

Recently, research in the liver tissue engineering domain has focused on the reproduction and long-term maintenance of hepatic functions *in vitro*. For example, it has been reported that the pore shape and size in printed structures significantly influenced hepatocyte growth (Sainz et al., 2009; Lewis et al., 2018). Additionally, it has been known that tissue-specific environmental factors are critical for preserving inherent cell morphologic properties, functions, and phenotypes (Flynn, 2010; Sellaro et al., 2010; Pati et al., 2014; Han et al., 2019). Accordingly, it is necessary to develop bioinks that can be tuned precisely and provide the cells with physiological properties similar to those of the *in vivo* microenvironment (Ma et al., 2020; Taymour et al., 2021). Hence, approaches have been pursued to fabricate 3D equivalent liver tissues with the long-term maintenance of high viability and functions using advanced bioinks (Taymour et al., 2021).

### 5.1 Drug Screening and *in vitro* Disease Models

Currently, there exists a need to develop a drug screening platform that can realize more accurate predictions. Recently, it has become mainstream to develop platforms that integrate 3D liver tissue constructs and microfluidic technology for drug screening (Snyder et al., 2011; Bhise et al., 2016; Lee et al., 2019). Accordingly, a 3D-printed *in vitro* liver model that can emulate the *in vivo* microenvironment has been attracting increased attention (Ma et al., 2016; Mao et al., 2020b, Mao et al., 2020c; Taymour et al., 2021; Xie et al., 2021; Yang et al., 2021). Yu et al. (2019) established a functional *in vitro* liver tissue model using DLP-based printing with LdECM and GelMA hybrid bioinks, which contained human induced pluripotent stem cell (hiPSC)-derived hepatocytes (hiPSC-Heps). This approach enabled the rapid fabrication of high-resolution microscale liver tissue constructs. In addition, the LdECM-based bioprinted construct exhibited high cell viability, similar to the collagen-based construct. Moreover, it provided a liver-specific microenvironment unlike collagen, improved the maturation of hepatocytes, and formed larger cellular aggregates. As a result, compared with the collagen-based construct, many larger positive staining areas of E-cadherin were observed, and the expression of liver maturation markers (TTR and albumin) was increased in the LdECM-based bioprinted constructs. Mazzocchi et al. (2019) independently developed a 3D co-culture liver model consisting of primary human hepatocytes and hepatic stellate cells using a methacrylated collagen I–thiolated HA hybrid bioink. The bioprinted construct maintained high cell viability and produced albumin and urea over a 2-week period. Moreover, the resulting construct exhibited a physiologically appropriate response to acetaminophen (APAP) (e.g., albumin, urea, alpha GST, LDH activity), which indicated that the printed liver tissue model has potential applicability in drug screening.

Moreover, a patient-specific liver tissue model is required to predict drug response more accurately. In another study, Xie et al. (2021) established patient-specific hepatocellular carcinoma (HCC) models *via* printing using gelatin–alginate bioinks. The bioprinted HCC models exhibited high survival rates (>80%) even after 30 days. Furthermore, the bioprinted models maintained stable expressions of AFP, a specific marker of HCC, genetic alterations, and stable expression profiles of all patient tumors in the long term. Subsequently, the authors created patient-derived xenograft (PDX) models by transplanting cells under the skins of BALB/C-nu mice, which were isolated from the bioprinted HCC. This experiment demonstrated that both AFP and Ki-7 yielded different expressions in PDX models and patient HCC specimens. Additionally, patient-specific bioprinted HCC models were used to evaluate the efficacy of various drugs (Sorafenib, Regorafenib, and Apatinib). Interestingly, each patient-derived model responded differently depending on the types of drugs and their concentration. In another research study, a patient-specific 3D intrahepatic cholangiocarcinoma (ICC) model was established. Mao et al. (2020c) developed a 3D in vitro tumor model via printing with a gelatin‐alginate‐matrigel hybrid bioink, which included patient‐derived primary ICC cells. The grid structure of the printed model was more conducive to tumor cell growth. As a result, it maintained high cell viability with continuous proliferation and induced the formation of spherical cell clusters. Remarkably, 3D-printed tumor models featured higher upregulated tumorigenic phenotypes than 2D culture models, such as the tumor marker (CA19-9 and CEA), cancer SC (EpCAM and CD 133), and invasive and metastatic marker expressions (MMP9 and MMP2 protein). Furthermore, the 3D-printed tumor model was used to study the epithelial–mesenchymal transition (EMT) phenomenon. It demonstrated that the expressions of EMT regulatory protein markers (E-cadherin and N-cadherin) changed in a similar manner to the *in vivo* phenomenon. Additionally, ECM components (HA, LN, P III P, and CIV) accumulated at larger quantities in the 3D-bioprinted tumor model than the 2D culture model, recapitulating the *in vivo*-like tumor microenvironment. Moreover, the bioprinted tumor yielded higher expressions of both liver damage and functional markers than those yielded by 2D models.

A research team developed a drug screening platform suitable for observing long-term drug responses, wherein 3D-bioprinted constructs were included in a microfluidic-based bioreactor. Bhise et al. (2016) produced spherical liver constructs with the use of GelMA, which contained hepatic spheroids. They cultured 3D hepatic spheroid-laden spherical liver tissue constructs in a perfusable bioreactor and demonstrated that the spheroids survived and maintained their functions for over 30 days. In terms of functionality, the printed construct maintained the secretion of liver-specific proteins (albumin, A1AT, transferrin, and ceruloplasmin) over time and the expression of the liver-specific genes (CK 18, MRP2, and ZO-1) for 30 days. The authors used the 3D-bioprinted liver on a chip to study the response of the chip to APAP. As a result, they demonstrated that both the cellular metabolic activity and the expression of the liver-specific gene (MRP2) were significantly decreased. Moreover, the secretion of liver-specific proteins (albumin, A1AT, transferrin, and ceruloplasmin) decreased over time in the cases of APAP-treated cultures.

**FIGURE 7:**
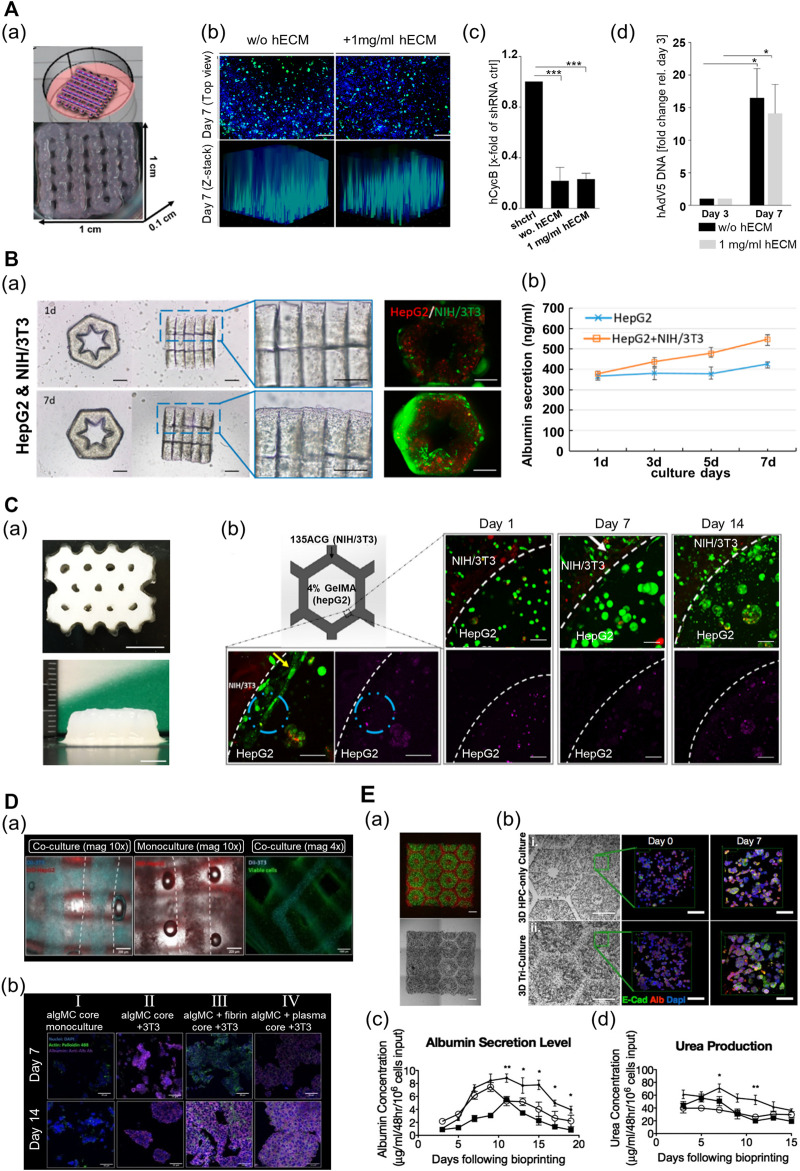
Multi-component hydrogel bioink-based 3D-printed liver tissue models. **(A)** 3D-printed liver tissue for infection and transduction studies. (a) 3D-printed liver tissue construct with HepaRG cell-laden hybrid alginate–gelatin–human extracellular matrix (hECM). (b) Comparison of AAV2.6 transduction and distribution within the multi-component bioink-based 3D-printed liver tissue construct with hECM or without hECM 7 days after printing. Blue: nuclei, green: AAV vectors. Scale bar: 200 μm. (c) Comparison of shRNA-mediated hCYcB RNA knockdown within the printed liver tissue construct with hECM or without hECM 7 days after printing. Results are shown as mean. ***p≤0.001. (d) Comparison of adenovirus replication within the 3D-printed liver tissue construct with hECM or without hECM. Reproduced with permission from Hiller et al. (2018). **(B)** 3D lobule-like microtissue assembled with co-culture 3D-printed micromodules. (a) The proliferation of the cells within the lobule-like 3D-printed microtissue for 7 days. (b) The evaluation of albumin secretion of HepG2 cells in mono-cultured 3D microtissue and co-cultured 3D microtissue for 7 days. Reproduced with permission from Cui et al. (2019). **(C)** 3D-printed bicellular liver tissue construct with in-direct co-culture honeycomb structure. (a) Top and side view of the embedded-printed structures with a height of 6.8 mm. Scale bars: 5 mm. (b) Changes in growth, proliferation, and morphology of cells embedded in compartmentalized bicellular liver-mimetic construct over 2 weeks. Reproduced with permission from Wu et al. (2020). **(D)** 3D-printed core–shell strand liver tissue construct. (a) Visualization of cell distribution in core–shell structure [DIL-labeled NIH3T3 (cyan), DIO-labeled HepG2 (red)]. Scale bar: 1000 μm. (b) Evaluation of hepatic function for the presence of co-culture with supporting cells or ECM components in the printed construct (purple—albumin stained). Reproduced with permission from Taymour et al. (2021). **(E)** 3D-printed tri-culture liver tissue construct with biomimetic liver lobule-like pattern. (a) Images taken in fluorescence and brightfield channels revealing the patterns of fluorescently labeled hiPSC-HPCs (green) in 5% (wt/vol) GelMA and support cells (red) in 2.5% (wt/vol) GelMA containing 1% GMHA on day 0. Scale bar: 500 μm. (b) Comparison of aggregation and intercellular interaction of hiPSC-HPC in 3D HPC-only construct and 3D triculture construct on days 0 and 7 [albumin (Alb), E-cadherin (E-cad), and nucleus (Dapi)]. Scale bars: 500 μm in bright field 100 μm in fluorescent images. (c) Comparison of albumin secretion levels of hiPSC-HPCs in three different conditions over time. (d) Comparison of urea secretion levels of HPCs in three different conditions over time. Error bars represent SEM, and *n* = 3 for all data points. Reproduced with permission from Ma et al. (2016).

3D-bioprinted liver disease models can assist the study of pathogenesis and progression of liver disease and drug development. The research has recently focused on providing the liver tissue microenvironment in diseased states with cells to achieve cellular reactivity and behaviors similar to those *in vivo*. A research team developed 3D-printed liver tissue suitable for virus biology research using an alginate-based multicomponent bioink (alginate–gelatin–human dECM) containing HepaRG cells (Figure 7A) (Hiller et al., 2018). The printed liver construct with dECM prolonged cell viability and enhanced hepatic functionality (albumin secretion and CYP3A4 activity). Additionally, as the resulting liver tissue construct featured geometrical strength, adeno-associated virus vectors could penetrate the cells embedded in the printed construct. As a result, the endogenous target gene, human cyclophilin B, was efficiently knocked out by ribonucleic acid interference (RNAi)-mediated silencing. Furthermore, the same team successfully infected the bioprinted construct with the human adenovirus 5 (hAdV5). As a result, the viral deoxyribonucleic acid was appropriately replicated over time in the resulting construct.

In another study, researchers created physiologically relevant 3D *in vitro* liver cirrhosis models using multicomponent bioinks with controllable stiffness. Liver fibrosis or cirrhosis is one of the pathophysiological processes caused by HCC, which results in the stiffening of the liver ECM. As a conventional model for liver fibrosis or cirrhosis does not feature a clinical stiffness range and recapitulates the liver-specific microenvironment for HCC, it has been challenging to study HCC progression in diseased states (Liu et al., 2015; Ma et al., 2018). Ma et al. (2018) printed a liver cancer tissue model using DLP-based 3D bioprinting technology with a collagen I hydrogel and photocrosslinkable LdECM-based bioink, to study the progression of HCC in the cirrhotic liver microenvironment. The researchers engineered a biomimetic liver cancer tissue platform composed of a LdECM-based hexagon, which included three different stiffnesses and collagen I-based septa regions within the liver nodules. The increased invasive potential of HepG2 under cirrhotic matrix stiffness at the genetic level and the remarkable invasive behavior of HeG2 into the fibrous septa from the stiff matrix were identified through this platform.

### 5.2 Liver Regeneration Models

3D-printed liver tissue models are promising tools and could serve as therapeutics in regenerative medicine. Moreover, in the case of severe liver damage, external intervention is essential to promote the regeneration of the liver. Therefore, efforts are focused on producing implantable 3D liver tissue models that can efficiently function *in vivo* in the long term. In terms of the scalability of printed tissue constructs, a researcher team established an approach to produce larger tissues *via* cell-based assembly among the functional liver tissue blocks. Cui et al. (2019) used GelMA to fabricate a lobule-like micromodule structure (Figure 7B). The printed micromodule had a hexagonal geometry with an inner radial pattern cavity that served as a vessel-like structure for mass transfer. The authors then assembled several micromodules coated with a GelMA bioink containing fibroblasts to create larger lobule-like microtissues by using noncontact micromanipulation techniques. The structural integrity of the assembled microtissues was improved with the widespread proliferation of the fibroblast along the surface of the tissue. Moreover, the assembled microtissues, which consisted of HepG2 and fibroblast cells, exhibited higher viability and hepatic functions (albumin secretion and urea synthesis) than those without fibroblasts. In another study, using the same printing method, the research team fabricated a 3D liver tissue construct with an inner-gear-like structure using a GelMA–LdECM hybrid bioink that contained human-induced hepatocytes (hiHeps) (Mao et al., 2020b). The author realized an improvement in the cell function by increasing the body surface area of the printed construct through the inner-gear-like structure. The resulting construct exhibited higher shape fidelity and enhanced cell viability and proliferation, as compared with the gelatin-based tissue construct without LdECM. Additionally, in the construct printed using the hybrid bioink, liver-specific function (secretion of albumin and urea) was enhanced, and the size of the cells was increased. However, the hepatic functionality of the printed construct decreased after 5 days in culture, and cell viability was lowered after 7 days in culture. Thus, further advances are required to maintain the function and viability of the cells for a long period in the printed structure.

Recently, to maintain long-term hepatic functionality and generate larger tissue constructs, hybrid 3D-printed liver constructs that enable the co-culture of hepatocyte and nonparenchymal cells have been developed using various multicomponent bioinks. Wu et al. (2020) fabricated a liver lobule-mimetic honeycomb structure using extrusion-based printing with a GelMA and ACG hybrid bioink (Figure 7C). The framework was formed with a honeycomb lattice pattern using an ACG bioink, and the cavity within the framework was filled with GelMA. The printed construct had good structural fidelity, and a construct up to 6.8 mm in height could be manufactured using the embedded printing technique. HepG2-laden monocellular constructs composed of GelMA maintained high cell viability and supported the formation of cell clusters and high cell proliferation for >7 days. In addition, the 3D construct enhanced the liver-specific function (albumin production), as compared with the 2D culture. Moreover, the HepG2/NIH/3T3 bicellular co-culture system improved the viability and functions of HepG2 for >14 days. A research team recently established a printing process that produced a heterogeneous liver-mimetic structure by simultaneously printing with two types of multicomponent bioinks containing different cells, where two types of cells were placed in a pattern that resembled an *in vivo* structure. Taymour et al. (2021) used a cell-laden algMC bioink with ECM components (fibrin or blood plasma, and matrigel) and an extrusion-based printing technique combined with a co-axial nozzle to fabricate a core–shell strand scaffold-shaped liver-mimetic tissue, wherein HepG2 and fibroblasts were compartmentalized into the core and shell, respectively (Figure 7D). As this construct exhibited appropriate mechanical stability, additional stabilization was not required. Thus, there was no requirement to consider the effects of chemical composition or different materials that could adversely affect cell behavior. Moreover, cell-laden constructs with bioactive components maintained high cell viability, exhibited improved cellular behavior, and influenced the formation of cellular morphology. In the construct with bioactive components, hepatocytes formed clusters featuring a structure with more prominent actin-based filaments of cells, and both the number and size of the cell clusters increased. The proliferation rate of HepG2 increased for 10 days, compared with that in the construct without the bioactive components. The fibroblasts also had cell-specific morphologies, and the formation of the cell network improved in the construct with ECM components. Furthermore, a co-culture system with indirect intercellular interactions between HepG2 and fibroblasts enhanced the hepatocyte-specific markers.

Vascularization is essential in the fabrication of engineered liver tissues to recapitulate the liver tissue microenvironment and promote hepatic functions. Moreover, it supports effective *in vivo* transplantation and scalability of the 3D-bioprinted liver tissues. In another study, two types of multicomponent bioinks were utilized to produce pre-vascularized liver tissue constructs with a patterned vascular structure. Ma et al. (2016) employed the DLP-based 3D bioprinting technique with GelMA and a mixture of GMHA and GelMA to fabricate a hexagonal in vitro liver tissue model as the basic unit of the liver, similar in size to hepatic lobules (Figure 7E). This printed structure provided a biomimetic liver microenvironment, wherein the hepatocytes and supporting cells were compartmentalized. Additionally, the matrix in the printed construct had a similar stiffness to that of healthy liver tissues. The human induced pluripotent stem cells (hiPSC)-derived hepatic progenitor cells (hiPSC-HPCs) in the printed model exhibited in vivo-like morphological characteristics over time, which were better than those of the 2D and HPC-only construct. Moreover, the resulting printed tissue exhibited high liver-specific functions. It maintained the liver-specific functions of cells in the printed model (such as albumin secretion and urea production) at a significant level longer than the other controls. Furthermore, key CYP enzymes (CYP3A4, CYP2C9, CYP2C19, CYP2B6, and CYP1A2), which are key enzymes in liver drug metabolism, were strongly expressed in the printed model; this indicated enhanced drug induction potential better than those of the other controls.

The 3D-printed liver tissue models introduced above were not transplanted *in vivo*. Conversely, there exists a case wherein a 3D liver tissue model printed with multicomponent bioinks was transplanted (Yang et al., 2021). Yang et al. (2021) bioprinted liver tissue constructs with alginate–gelatin hybrid bioink that contained HepaRG cells. To emulate physiological conditions, the authors induced the differentiation of HepaRG using dimethyl sulfoxide to obtain a 3D-printed liver tissue construct composed of two types of cells (matured hepatocytes and cholangiocytes). The bioprinted construct featured a comparable level of the liver-specific gene expression (ALB, CK18, AAT, MRP2, and transferrin), induction of liver-specific transcription factors (FOXA2 and HNF4A), synthesis of liver-specific protein (human albumin, alpha-1 antitrypsin, and factor VII), and detoxification activity (CYP1A2 and CYP3A4) to the primary human hepatocyte. Furthermore, it showed an appropriate level of other hepatic functions (glycogen storage and ICG uptake and release). Subsequently, the authors transplanted the 3D-printed liver tissue construct into the abdominal cavities of a $Fah^{-/-}Rag2^{-/-}$ (F/R) liver injury mouse model. The transplantation of the 3D-printed liver tissue construct increased the synthesis of liver-specific proteins (mouse albumin, total protein, and prealbumin). In addition, it reduced the serum level of the biomarkers related to liver injury (ALT, TBIL, DBIL, GGT, and ALP) and downregulated the serum levels of amino acids. Remarkably, the transplanted liver construct was fully neovascularized *in vivo* and still retained the ability to produce human liver-specific protein (human albumin, alpha-1 antitrypsin, factor VII, and factor IX) and drug metabolites (human-specific debrisoquine) after 4 weeks of transplantation. Multicomponent bioink-based 3D-printed liver tissue models for drug screening and liver disease models are summarized in Table 2 and for liver regeneration models are summarized in Table 3 and Table 4.

**TABLE 2:** Single structure of multicomponent bioink-based 3D-printed liver tissue models for drug screening and liver disease models.

Engineered construct	Applied material	Cells/cell line	Major highlights of the study	Ref.
Hexagonal lobular structure	Liver dECM + GelMA	hiPSC-Heps	Establishment of efficient fabrication of biomimetic liver tissue microarray with photocrosslinkable liver-specific bioink	Yu et al. (2019)
Four-spoke structure	Methacrylated collagen type I + thiolated hyaluronic acid	Primary human hepatocyte liver stellate cell	Development of the printable and ECM-like collagen-based multicomponent bioink that can maintain long-term hepatic functions	Mazzocchi et al. (2019)
3D grid	Gelatin + alginate	Primary hepatocellular carcinoma cells	Establishment of patient-specific 3D-bioprinted hepatocellular carcinoma models for drug testing	Xie et al. (2021)
3D grid	Gelatin + alginate + matrigel	Primary intrahepatic cholangiocarcinoma cells	Development of patient-specific 3D-bioprinted intrahepatic cholangiocarcinoma model for drug testing	Mao S. et al. (2020)
Spherical structure	GelMA	HepG2/C3A	Development of 3D-bioprinted liver-on-a-chip platform integrated with bioreactor enabling long-term maintenance of liver functions	Bhise et al. (2016)
3D grid	Gelatin + alginate + human lung dECM	HepaRG	Fabrication of 3D-printed liver tissue model that allowed extensive transduction viral vectors	Hiller et al. (2018)
Lobule structure	Liver dECM + GelMA	HepG2	Development of 3D-bioprinted liver disease model with cirrhosis matrix stiffness enabling the implementation of liver cancer cell behavior	Ma et al. (2018)

**TABLE 3:** Single structure of multicomponent bioink-based 3D-printed liver tissue models for liver regeneration models.

Engineered construct	Applied material	Cells/cell line	Major highlights of the study	Ref.
Gear shape with inner cavity	GelMA	HepG2 NIH/3T3 fibroblast	Development for multicellular co-culture 3D liver microtissues assembled with liver-lobule like micromodules	Cui et al. (2019)
Inner gear-like structure	Liver dECM + GelMA	hi-Hep	Development of liver-specific bioink compatible with DLP-printing strategy	Mao Q. et al. (2020)
3D grid	Gelatin + alginate	HepaRG	Development of implantable 3D-printed liver organoid performing systematic liver functions both *in vitro* and *in vivo*	Yang et al. (2021)

**TABLE 4:** Hybrid structure of multicomponent bioink-based 3D-printed liver tissue models for liver regeneration models.

Engineered construct	Applied material		Cells/cell line	Major highlights of the study	Ref.
	Material #1	Material #2			
3D honeycomb structure	Alginate + cellulose nanocrystal + GelMA	GelMA	HepG2, NIH/3T3 fibroblast	Development of compartmentalized bicellular 3D-printed liver-lobule like construct performing enhanced liver functions	Wu et al. (2020)
3D grid (core–shell strand)	Alginate + methylcellulose + matrigel	Alginate + methylcellulose + fibrinogen or blood plasma	HepG2, NIH/3T3 fibroblast	Development of co-culture 3D-printed liver tissue with core–shell structure enhancing liver functions	Taymour et al. (2021)
Lobule structure with vascular structure	GelMA	GMHA + GelMA	hiPSC-HPCs, HUVECs, ADSCs	Development of tri-culture 3D-printed liver tissue model mimicking liver lobule pattern *in vivo* and enhancing liver functions	Ma et al. (2016)

## 6 Conclusion and Future Perspectives

In liver tissue engineering, the goal is the construction of 3D biomimetic liver tissue models that exhibit functionality resembling that of native liver tissues for applied research, such as disease pathogenesis, drug and toxicity metabolism, and regenerative medicine. Compared with traditional fabrication methods, 3D bioprinting makes it possible to precisely deliver and position the bioinks containing different cells and ECM components, thus realizing the reconstruction of the complex structure of liver tissues. Thus, 3D bioprinting techniques have been extensively utilized in the fabrication of liver tissue models.

For 3D bioprinting, the most significant component to be considered is the bioink. The bioink should protect cells against the applied force and exhibit high resolution and shape fidelity during printing. After printing, the bioink should possess good mechanical characteristics to maintain the structural stability of the 3D-printed cell-laden construct; it is also essential to provide the cells with a biocompatible environment for tissue development. However, existing bioinks composed of a single biomaterial cannot possess both high printability and biological functionality. Therefore, the use of multicomponent bioinks, which are capable of preserving the strengths of each biomaterial, has become an emerging trend. In liver tissue engineering, significant progress has been achieved in terms of bioprinting 3D functional liver tissues using various multicomponent bioinks. However, certain issues in terms of selecting the biomaterials and designing multicomponent bioinks for the 3D bioprinting of liver tissues still exist, and these need to be addressed.

A comprehensive understanding of the liver ECM is considered necessary to prepare multicomponent bioinks that emulate the liver ECM. Various components in the liver ECM are involved in determining cell morphology, cell behavior, and cell function *via* interactions with the cell. As it is difficult to replicate such a complex dynamic relationship between the liver ECM and cells using a single natural biomaterial, several research teams have designed multicomponent bioinks with liver dECM. However, it is difficult to prove the biostability and effectiveness of dECM as chemically undefined components remain in the liver ECM. Furthermore, there are differences in the dECM elements attributed to differences in the decellularization protocols among research teams. Therefore, it is necessary to establish a decellularization protocol that can thoroughly remove the cellular components, while preserving the cues from the liver-specific ECM crucial for liver development insofar as possible; this should also be supplemented with continued research focused on defining ECM components. In addition, with the development of genetic engineering technology, it is anticipated that implantable liver tissue constructs will be fabricated; these can subsequently be applied in clinical treatments with dECM prepared from xenogeneic or allogeneic tissues, wherein the immunogenic genes will be removed. To support this, a research team utilized porcine tissue as a material for dECM to print bone tissues, wherein the immune-rejection gene, α-1,3-galactose (α-1,3-gal), was knocked out (Gao et al., 2021).

It is necessary to establish various crosslinking strategies that are both biocompatible and rapid for preparing multicomponent bioinks with high printability. In particular, the photocrosslinking mode is extensively used in tissue engineering, as it is a rapid crosslinking system, straightforward to control, and applicable to various printing techniques. Numerous multicomponent bioinks, including photosensitive biomaterials, have also been applied in liver tissue bioprinting. However, most of the photosensitive biomaterials extensively used in this process are crosslinked under UV conditions, which can potentially induce cytotoxicity. Hence, it is important to consider a photocrosslinking method that can be employed under visible light, as this would impose a less detrimental effect on cells. In addition, it is expected that supramolecular bioinks can be applied in bioprinting liver tissues as this does not require additional stimuli or substances with potentially detrimental effects on cells. Therefore, it is anticipated that various types of multicomponent bioinks with biocompatible and rapid crosslinking modes can be conducive to the scalability of bioprinted structures.

Along with the development of multicomponent bioinks for the fabrication of fully functional liver tissue models, advances in related technologies are essential, such as SC, printing, microfluidic, and micromanipulation technologies. Medical trends have shifted toward personalized medicine that has facilitated the development of patient-specific liver tissue models using patient-specific SCs (mesenchymal or iPSCs). Patient-specific liver tissue models can be conducive to personalized medicine, as the treatments and prevention methods are specifically tailored considering genetic, physical, and environmental differences between patients. Hence, it is necessary to establish SC technologies to supply patient-derived SCs necessary for developing personalized liver tissue models in a stable manner. Additionally, a bioreactor based on microfluidic technologies is essential for the long-term culture of printed liver tissue models. There is also a requirement to continuously develop liver tissue fabrication strategies with advanced bioprinting techniques. In terms of applications, the conjugation of 3D-printed liver tissues and microfluidic systems integrated with biosensors could enable real-time monitoring, thus introducing a new paradigm to the drug screening platform. Furthermore, micromanipulation technology could serve as one of the strategies to build volumetric liver tissue constructs (Ip et al., 2018; Ayan et al., 2020). This technology enables the control of functional liver tissue blocks to assemble the blocks according to the predefined design; this is expected to efficiently produce volumetric liver tissues. Therefore, cooperation between various disciplines will assist the achievement of significant progress in liver tissue engineering.

In summary, we introduced multicomponent bioinks for 3D printing applications. These multicomponent bioinks could be fabricated with the use of various fabrication methods and materials by considering their materials characteristics, mechanical properties, and functionality. Notably, 3D bioprinting with multicomponent bioinks has provided a unique opportunity to research *in vitro* liver tissue models. Versatile bioprinting techniques offer reproducible methods to create a sophisticated 3D cell-laden structure, as compared with conventional tissue engineering methods. Moreover, the development of multicomponent bioinks has enabled the production of functional tissues with complex structures by improving the biofunctionality and mechanical stability of single-component bioinks. In this manner, the various attempts adopted to recapitulate the liver microenvironment by designing microstructures and matrix properties similar to those of native liver tissues have extended the maintenance of cell viability, functions, and cellular phenotype. However, it is still challenging to fabricate *in vitro* 3D liver tissues that mimic the biochemical and biophysical characteristics and microstructure of native liver tissues. Thus, further significant efforts are required to manufacture clinical scale, mechanically robust, 3D-bioprinted equivalent liver tissues for human treatment and drug screening. Therefore, various multicomponent bioinks consisting of new biomaterials and new material combinations as well as ideal bioprinting strategies will be developed to address these limitations and allow for continued significant advancements in liver tissue engineering.
